# Real‐time imaging of respiratory effects on cerebrospinal fluid flow in small diameter passageways

**DOI:** 10.1002/mrm.29248

**Published:** 2022-04-10

**Authors:** Johannes Töger, Mads Andersen, Olle Haglund, Tekla Maria Kylkilahti, Iben Lundgaard, Karin Markenroth Bloch

**Affiliations:** ^1^ Department of Clinical Sciences Lund, Diagnostic Radiology Lund University, Skåne University Hospital Lund Sweden; ^2^ Philips Healthcare Copenhagen Denmark; ^3^ Lund University, Lund University Bioimaging Center Lund Sweden; ^4^ Department of Medical Radiation Physics Lund University Lund Sweden; ^5^ Department of Experimental Medical Science Lund University Lund Sweden; ^6^ Wallenberg Centre for Molecular Medicine Lund University Lund Sweden

**Keywords:** cerebrospinal fluid, CSF, flow, magnetic resonance imaging, MRI, real‐time

## Abstract

**Purpose:**

Respiration‐related CSF flow through the cerebral aqueduct may be useful for elucidating physiology and pathophysiology of the glymphatic system, which has been proposed as a mechanism of brain waste clearance. Therefore, we aimed to (1) develop a real‐time (CSF) flow imaging method with high spatial and sufficient temporal resolution to capture respiratory effects, (2) validate the method in a phantom setup and numerical simulations, and (3) apply the method in vivo and quantify its repeatability and correlation with different respiratory conditions.

**Methods:**

A golden‐angle radial flow sequence (reconstructed temporal resolution 168 ms, spatial resolution 0.6 mm) was implemented on a 7T MRI scanner and reconstructed using compressed sensing. A phantom setup mimicked simultaneous cardiac and respiratory flow oscillations. The effect of temporal resolution and vessel diameter was investigated numerically. Healthy volunteers (*n* = 10) were scanned at four different respiratory conditions, including repeat scans.

**Results:**

Phantom data show that the developed sequence accurately quantifies respiratory oscillations (ratio real‐time/reference *Q*_R_ = 0.96 ± 0.02), but underestimates the rapid cardiac oscillations (ratio *Q*_C_ = 0.46 ± 0.14). Simulations suggest that *Q*_C_ can be improved by increasing temporal resolution. In vivo repeatability was moderate to very strong for cranial and caudal flow (intraclass correlation coefficient range: 0.55–0.99) and weak to strong for net flow (intraclass correlation coefficient range: 0.48–0.90). Net flow was influenced by respiratory condition (*p* < 0.01).

**Conclusions:**

The presented real‐time flow MRI method can quantify respiratory‐related variations of CSF flow in the cerebral aqueduct, but it underestimates rapid cardiac oscillations. In vivo, the method showed good repeatability and a relationship between flow and respiration.

## 1 INTRODUCTION

Cerebrospinal fluid (CSF) is critical for brain health and mechanical protection. Furthermore, recent research suggests that CSF is involved in circulation and clearance of waste products in the brain through the glymphatic system. Since the initial proposal by Iliff et al in 2012,^1^ glymphatic clearance has been shown in rodents^2^ and higher mammals,^3^ and recent evidence suggests a similar mechanism in humans.^4^, ^5^, ^6^ However, the existence and function of the glymphatic system in humans is not robustly proven, and methods for accurate quantification of CSF flow in humans may help elucidate these mechanisms.^6^, ^7^ Furthermore, such methods could potentially become a tool for risk stratification, diagnosis, and follow‐up in diseases with intracranial pressure disturbances such as normal pressure hydrocephalus,^8^, ^9^, ^10^, ^11^ congenital malformations,^12^ and cognitive diseases.^13^

Flow of CSF has been quantified using MRI flow methods,^14^, ^15^ showing a correlation between CSF flow and the heart beat^16^, ^17^ and variation with the circadian rhythm.^18^, ^19^ According to the Monro‐Kellie doctrine, fluctuations in the intracranial CSF volume help to maintain constant intracranial pressure, as respiratory changes in thoracic pressure drive changes in venous return from the brain. Recent results have shown that respiration is the dominant regulator of aqueduct CSF flow in humans,^20^, ^21^, ^22^ opening up a new avenue for investigation of CSF flow physiology and pathophysiology. Early works on CSF flow used cardiac gating,^10^, ^11^, ^18^, ^19^ implicitly assuming that CSF flow only depended on cardiac motion, and therefore were insensitive to respiratory effects. Because breathing is in general nonperiodic, it is necessary to use *real‐time* methods, defined in this context as dynamic imaging that does not use cardiac gating.

Although CSF flow can be measured in multiple locations, the present work focuses on the cerebral aqueduct. The aqueduct, with a diameter of 1–3 mm, is a central pathway for CSF circulation, connecting the third and fourth ventricles of the brain. Due to its location, aqueduct flow reflects the net CSF production in the lateral and third ventricles. Furthermore, the narrow diameter leads to an increased dynamic range of velocities for measurement, potentially amplifying changes in CSF flow dynamics.

Several recent studies have used real‐time flow MRI to study respiratory CSF flow. An early study used a pencil‐beam excitation to measure flow and displacement of the brain and CSF in a single image line over time, similar to an M‐mode echocardiogram.^23^ Chen et al^20^ used a simultaneous multislice velocity‐encoded EPI sequence covering the ventricles, aqueduct, and foramen of Monro with 2.5 × 2.5 mm in‐plane resolution and temporal resolution of 80 ms, and demonstrated increased CSF flow during deep breathing. Yildiz et al^24^ used an echo‐planar imaging (EPI) method to quantify CSF flow in the foramen magnum and along the spinal cord with an in‐plane spatial resolution of 2.5 × 2.5 mm and temporal resolution 50 ms, including a phantom experiment to validate the accuracy of their technique. Because these sequences have a low spatial resolution compared with the aqueduct diameter, large errors are likely.^25^ Dreha‐Kulaczewski et al^26^ used phase contrast with spatial resolution 1.2 × 1.2 mm and temporal resolution 135 ms, and Aktas et al^27^ used a spatial resolution of 0.75 × 0.75 mm and temporal resolution of 125 ms, although without validation experiments. To the best of our knowledge, no study to date has presented a phantom validation of real‐time flow imaging in diameters comparable to the aqueduct. Furthermore, to assess the precision and practical use of the technique, there is a need to investigate the repeatability of in vivo measures of respiratory CSF flow.

Therefore, we aimed to (1) develop a real‐time flow imaging method with high spatial resolution and sufficient temporal resolution to capture respiratory CSF flow in the cerebral aqueduct, (2) validate the performance of the method in a phantom setup and numerical simulations mimicking flow conditions in the aqueduct including cardiac and respiratory oscillations, and (3) apply the method in healthy volunteers and investigate its repeatability and correlation with different respiratory conditions.

## 2 METHODS

### 2.1 Magnetic resonance imaging equipment and real‐time sequence

All MRI data were acquired using a 7T MRI scanner (Achieva; Philips Healthcare, Best, the Netherlands) and a 2‐channel transmit, 32‐channel receive head coil (Nova Medical, Wilmington, MA, USA).

Flow quantification was performed using a single‐slice radial gradient‐echo sequence with a through‐plane flow‐encoding gradient and RF and gradient spoiling. Sequence parameters are summarized in Table 1. For the phantom validation, a range of spatial resolutions were acquired, whereas for in vivo scans, spatial resolution was fixed at 0.6 mm. The radial k‐space acquisition used a golden‐angle scheme,^28^ such that the angle increment between subsequent readout directions was 111.246°. This ensures that each spoke contributes an optimal additional coverage of k‐space compared with previous spokes, which gives flexibility in the reconstructed temporal resolution. Golden‐angle ordering also ensures that artifacts from undersampling are incoherent in space and time, which provides favorable conditions for sparsity‐based image‐reconstruction methods.^28^, ^29^ For each radial spoke, two acquisitions were performed with alternating signs of the velocity‐encoding gradient.

**TABLE 1:** Summary of MRI sequence parameters for phantom and in vivo scans.

Parameter	Real‐time flow sequence	Gated reference flow sequence for phantom
Readout strategy	Golden‐angle radial	Cartesian
Gating	none, continuous real‐time acquisition	On respiratory component
VENC (cm/s)	15	15
TE (ms)	5.1	3.5
TR (ms)	10.5	7.9
Flip angle (°)	7	7
Bandwidth/pixel (Hz)	208	701
Slice thickness	5 mm	5 mm
FOV	240 × 240 mm	208 × 208 mm
In‐plane resolution (phantom)	0.5, 0.6, 0.7, 0.8, 0.9 mm (matrix 267 × 267–480 × 480)	0.5 mm (matrix 416 × 416)
In‐plane resolution (in vivo)	0.6 × 0.6 mm (matrix 400 × 400)	—
Scan time (min:s)	0:50	3:30
Radial spokes per frame	8	—
Reconstructed temporal resolution (ms)	168	67
Temporal segmentation factor	—	4

Parts of this work has previously been presented in a short conference abstract.^30^

### 2.2 Image reconstruction

**FIGURE 1:**
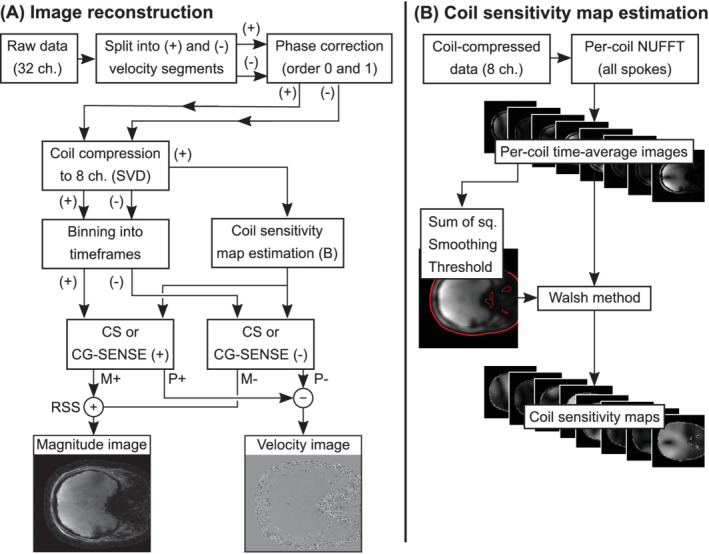
Image reconstruction framework. A, Image reconstruction method. B, Coil sensitivity map estimation. Abbreviations: CS, compressed sensing; CG, conjugate gradient; NUFFT, nonuniform fast Fourier transform; RSS, root sum of squares; SVD, singular value decomposition

The compressed‐sensing (CS) flow imaging reconstruction, based on earlier golden‐angle radial methods,^29^, ^31^ is summarized in Figure 1. To correct for eddy current and gradient delay errors, raw data were first corrected for spoke‐dependent echo shifts (typically referred to as first‐order phase correction), and a zeroth‐order phase correction was performed to make phases at *k* = 0 the same for all spokes.^32^ Coil compression^33^ was performed to eight virtual coils. The full data (acquired during 50 s) was used to form one image per coil using standard density‐compensated gridding techniques, and then the Walsh method^34^ was used to create coil sensitivity maps for the eight virtual coils.

Thereafter, binning into time frames was performed using eight radial spokes per frame, corresponding to 168 ms per frame. Images were reconstructed through an L1‐regularized parallel imaging and CS optimization formulation, similar to previous studies,^29^, ^31^, ^35^ as follows:

(1) $$\min_{x}{\left\|F_{r}\mathrm{Sx}-d\right\|}_{2}^{2}+\lambda {\left\|\mathrm{Tx}\right\|}_{1}.$$

The first term describes data consistency, with *F*_*r*_ describing the radial nonuniform Fourier transform, *S* the coil sensitivity maps, *x* the image time series to be reconstructed, and *d* the collected raw data. The second term describes the L_1_‐penalized temporal total variation regularization, where *λ* is a weighting factor between the two terms and *T* is the temporal total variation operator. The two velocity encodings were reconstructed separately (Figure 1A), and thereafter complex division was performed to find the phase difference representing the velocity.

Coil array compression, radial gridding, and the L1‐regularized CS reconstruction was performed using the Berkeley Advanced Reconstruction Toolbox (BART, v0.4.03)^36^ and *MATLAB* R2019a (The MathWorks, Natick, MA). The number of iterations was specified to 100, which was observed to provide convergence of the optimization process. The reconstruction parameter *λ* was set to three different values: 10^−3^, 10^−6^ and 10^−9^. Raw data were divided by the magnitude of the largest k‐space sample before image reconstruction, to make the effect of *λ* more consistent between subjects and data sets. Furthermore, the sparsity regularization was also turned off (corresponding to *λ* = 0), resulting in a conjugate‐gradient (CG) SENSE reconstruction.^37^ For in vivo scans, data from the full 50 s were reconstructed. For phantom data, only the first 20 s were reconstructed to save reconstruction time due to the large number of parameter combinations.

### 2.3 Phantom validation

**FIGURE 2:**
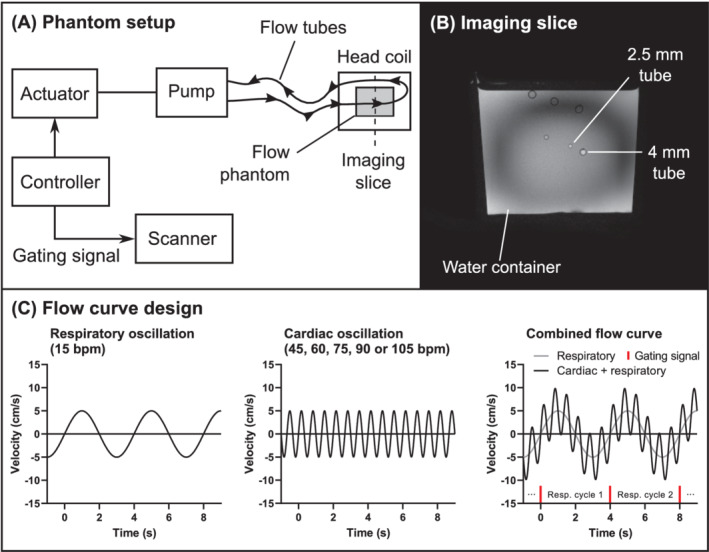
Phantom validation setup. A, An embedded controller controls an actuator and pump, which creates an oscillating flow in a set of plastic pipes submerged in a water container inside the head coil of the scanner. B, Image slice of the phantom setup, at the level where flow imaging was performed. Flow was measured in a 2.5‐mm and a 4‐mm tube. C, The phantom flow curve consisted of a respiratory oscillation at 15 cycles/min (period = 4 s), with a cardiac oscillation superimposed. The gating signal for the reference 2D‐flow scan was synchronized with the respiratory cycle

A phantom validation setup was constructed to investigate accuracy, as illustrated in Figure 2. An embedded controller (Arduino UNO rev. 3; Arduino, Monza, Italy) controls a servo motor that moves a ball‐screw actuator (RD‐55T‐12‐150 and Cool Muscle CM2‐X‐56B20C; Myostat, Newmarket, Canada). The actuator was connected to a piston, creating an oscillating flow in thin‐walled plastic tubes (diameters 2.5 and 4.0 mm) submerged in a 1.5‐L water container inside the head coil of the scanner. Gadolinium‐based contrast agent (Dotarem, Guerbet, France) was added to the water in the container, resulting in a T_1_ of approximately 1380 ms. Approximately 1 tablespoon of table salt was added to the container to improve B_1_ transmission homogeneity. There was no gadolinium added to the flowing water, which gave a T_1_ value of 3400 ms. The T_1_ values were measured using an inversion‐recovery turbo spin‐echo sequence.

The oscillating flow was programmed as a superposition of two sinusoidal components: a respiratory component at 15 cycles/min, and a cardiac component at 45, 60, 75, 90, or 105 cycles/min (Figure 2C). A gated flow sequence was used as reference (spatial in‐plane resolution = 0.5 mm, temporal resolution = 67 ms), with gating performed to the respiratory oscillation (15/min), so that the resulting velocity map captured a full 4‐s respiratory cycle and multiple cardiac cycles. For each cardiac frequency setting, the gated reference flow image was first acquired, followed by the real‐time sequence for all in‐plane resolution settings. The reference flow image was then re‐acquired to assess the stability of the setup.

**FIGURE 3:**
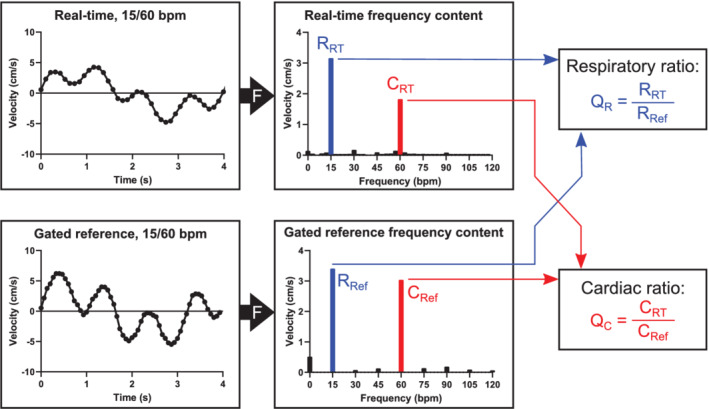
Analysis of phantom flow data. Flow curves were extracted from the real‐time (top) and gated reference (bottom) velocity maps. A fast Fourier transform–based spectrum analysis was performed (“F,” black arrows). The amplitudes of the respiratory components (blue) were extracted from the real‐time (R_RT_) and reference (R_Ref_) data sets, and their ratio *Q*_R_ was computed as an accuracy index. A similar analysis was performed for the cardiac component (C_RT_ and C_Ref_, in red color) to give the ratio *Q*_C_. A ratio close to 1 indicates accurate quantification in the real‐time data, and a ratio below 1 indicates that real‐time flow underestimates the oscillation

Analysis of the phantom data is shown in Figure 3. Respiratory and cardiac frequency components were extracted from the real‐time and reference flow data using a fast Fourier transform (FFT). The respiratory ratio *Q*_R_ was computed as the ratio of the amplitudes of the respiratory component in the real‐time and reference images. The same analysis was performed for the cardiac component, resulting in the cardiac ratio *Q*_C_. A ratio less than 1 indicates underestimation of the flow oscillation in the real‐time data; a ratio above 1 indicates overestimation; and a ratio equal to 1 indicates that the flow is quantified accurately.

### 2.4 In vivo scans

Healthy volunteers (*n* = 10, age 26 ± 2 years) were included to test the feasibility and repeatability of the method in vivo. The study followed the Helsinki declaration and was approved by the local ethical review board in Lund, Sweden. All volunteers provided written informed consent.

**FIGURE 4:**
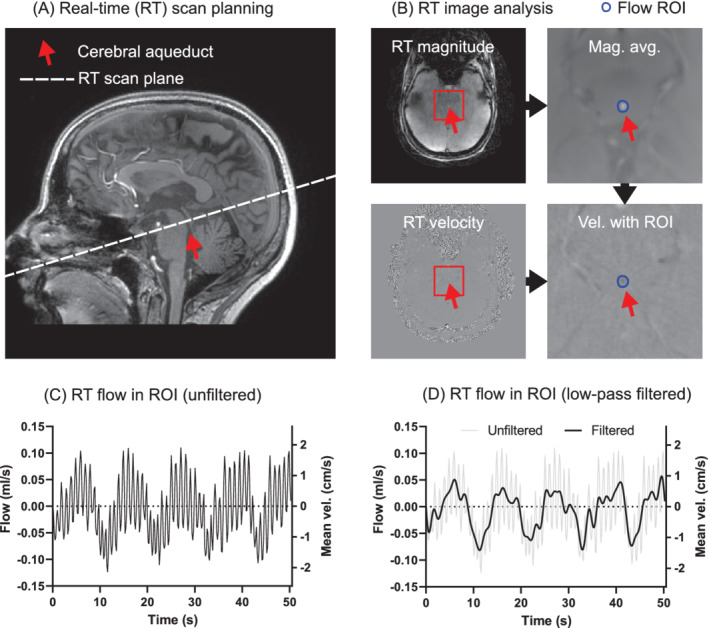
In vivo scan planning and analysis. A, The planning of the scan plane for aqueduct flow measurements. B, The method for placing the region of interest (ROI) using a time‐averaged magnitude image. C, The real‐time flow curve in the ROI. D, The way cardiac oscillations were filtered out to isolate respiratory variations

The MRI protocol included a whole‐brain 3D T_1_‐weighted localizer sequence (1‐mm isotropic). The proposed real‐time flow MRI sequence was planned perpendicular to the aqueduct in the localizer (Figure 4). A spatial resolution of 0.6 mm was used based on a balance between spatial resolution and reconstruction time, and the regularization factor *λ* was set to 10^−6^, as it visually balanced image quality and temporal smoothing. One data set was reconstructed with CG‐SENSE and CS with *λ* between 10^−2^ and 10^−9^ to assess sensitivity to *λ*. Each real‐time scan had a duration of 50 s. During the scans, the volunteers were asked to perform respiratory exercises as follows:

1. Free breathing at rest (“free”);
2. Deep breathing at a comfortable pace (“deep”);
3. Guided breathing with breath‐holds (BHs): 2‐s inspiration, 2‐s BH, 2‐s expiration, 2‐s BH, repeated for the duration of the scan (“guided‐BH”); and
4. Guided breathing without BHs: 2‐s inspiration, 2‐s expiration, repeated for the duration of the scan (“guided‐noBH”).

Exercise 1–4 were repeated immediately to assess repeatability, for a total of eight scans. The guided breathing exercises 3 and 4 were guided by projection of timed instructions onto a screen visible to the volunteer through a mirror.

### 2.5 In vivo analysis

Analysis of in vivo data is shown in Figure 4 using the medical image analysis software Segment v3.2 R8408 (Medviso, Lund, Sweden).^38^ The full FOV was cropped to a reduced area (approximately 50 × 50 mm) around the aqueduct to reduce storage and computation needs. For each subject, a temporal mean of the first real‐time image series (free breathing at rest) was created, and the aqueduct delineated manually. Thereafter, the delineation was transferred to all real‐time images in that subject. In cases with in‐plane motion due to vigorous breathing, the aqueduct was automatically tracked through the time series.

Phase background correction was performed by subtracting a first‐order polynomial fit to static tissue as detected by the software. Velocity anti‐aliasing was not performed, as no aliasing was seen in the images. The mean velocity (cm/s) and flow rate (ml/s) in the region of interest was then computed for each time frame. The flow for each timeframe was computed by multiplying the velocity in each pixel by the pixel area, and then summing over all pixels within the region of interest. To assess interobserver variation, two observers performed delineations independently. Agreement between observers was assessed using linear regression and intraclass correlation coefficients (ICCs). Similarity of delineations was quantified using the Dice coefficient.^39^

To isolate the respiratory effects on the flow curve and remove confounding cardiac oscillations, shown in phantom and numerical results subsequently to be underestimated using our method, low‐pass filtering was applied using a finite impulse response (FIR) filter, accepting all frequencies below 0.5 Hz, and rejecting all frequencies above 0.75 Hz. Passband ripple was set to 0.1 dB and stopband attenuation to 20 dB. The filter was applied twice with the second repetition reversed, resulting in a zero‐delay, zero‐phase filter, and a stopband attenuation of 40 dB.

For each scan, time frames were separated into positive flow (cranial, toward the top of the head) and negative flow (caudal, toward the feet) and summed to obtain the total positive and negative volume. The volumes were divided by the scan duration to obtain the flow in milliliters per minute. Net flow was computed by summing the flow in all timeframes and dividing by the scan duration. Flow data are presented as the mean of the two repeated measurements for each respiratory condition.

Resemblance of the CSF flow to the respiratory bellows signal was investigated. We hypothesized that CSF flow in the aqueduct is correlated with the respiratory bellows signal, but that there could be physiology‐induced time delay, shape differences, and amplitude differences between the two signals. To measure correlation between the two signals, we therefore used magnitude‐squared coherence,^40^ implemented in the “mscohere” function in *MATLAB* R2019a, shown in Supporting Information Figure S1A–C. The algorithm measures the coherence of the two signals as a function of frequency, based on an overlapping averaged periodogram method using eight segments and 50% overlap.^41^ The respiratory frequency was estimated as the frequency for which power spectral density of the respiratory bellows signal was highest, and the coherence value (between 0 for no coherence and 1 for full coherence) was reported.

### 2.6 Numerical model

**FIGURE 5:**
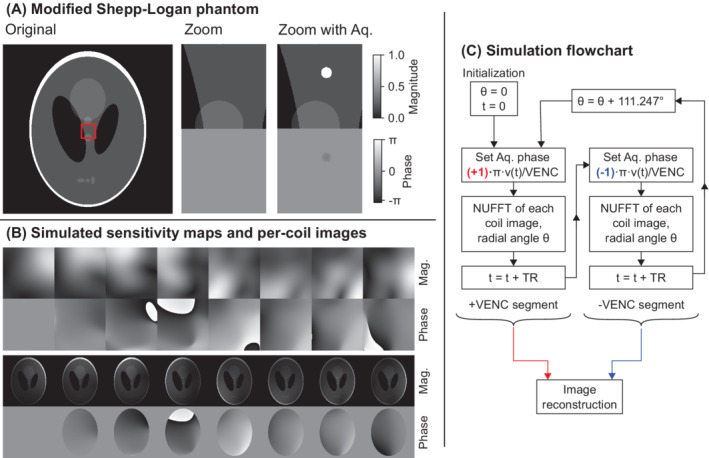
Numerical flow phantom. A, Modified Shepp‐Logan phantom with addition of an aqueduct with variable diameter *d*, including a time‐varying phase due to velocity. B, Simulated sensitivity maps and per‐coil images. C, The way k‐space data were generated. Abbreviation: VENC, velocity encoding

After reviewing phantom and in vivo data, a numerical model was created to gain additional insight into the imaging and reconstruction process (Figure 5). The numerical model was constructed similar to the MRXCAT framework,^42^ with synthetic coil maps and nonuniform FFT from the Berkeley Advanced Reconstruction Toolbox (BART, v0.4.03).^36^

A Shepp‐Logan numerical phantom^43^ was modified by adding an “aqueduct” (Figure 5A) with a temporally varying phase corresponding to the velocity in the phantom experiment. Multicoil acquisition with eight coils was modeled by multiplying the phantom image with modeled coil sensitivities^44^ (Figure 5B). A nonuniform FFT was performed to sample the Fourier transform of the image along radial spokes in k‐space for positive and negative velocity encodings separately (Figure 5C). Before each nonuniform FFT, simulation time was incremented by TR and the velocity re‐evaluated. The spoke angle was incremented by the golden angle every second TR. Simulations were performed without noise to simplify the analysis.

As baseline, we set *d =* 2.5 mm, heart rate = 60 bpm, TR = 10.5 ms, and 8 spokes per frame. Four numerical experiments were performed: (1) varying heart rate between 60 and 120 bpm in steps of 15 bpm; (2) varying TR between 2 and 10.5 ms in steps of 1 ms, resulting in temporal resolutions between 32 and 168 ms; (3) varying the aqueduct diameter between 2 and 10 mm in steps of 1 mm; and (4) varying the regularization factor *λ* between 10^−9^ and 10^−2^ with exponent steps of 0.5. After generating the multicoil time‐resolved k‐space data, image reconstruction was performed using the code used for MRI data.

### 2.7 Statistical methods

In vivo interobserver variability and repeatability was assessed using Bland–Altman analysis,^45^ linear regression, and ICC values (two‐way, single‐measurement agreement ICC). Following previous recommendations, ICC values below 0.3 were described as lack of agreement, between 0.31 and 0.50 as weak, between 0.51 and 0.70 as moderate, between 0.71 and 0.90 as strong, and between 0.91 and 1.00 as very strong agreement.^46^ Variability between different respiratory conditions was tested using analysis of variance. Group values are presented as mean ± SD.

## 3 RESULTS

### 3.1 Phantom validation

The phantom setup showed high stability for both the slow (pre‐real‐time scans: 3.02 ± 0.28 cm/s vs post‐real‐time: 2.99 ± 0.28 cm/s; bias 0.03 ± 0.02 cm/s or 1.0% ± 0.9%) and fast flow component amplitudes (pre‐real‐time: 2.76 ± 0.23 cm/s vs post‐real‐time: 2.72 ± 0.23 cm/s; bias 0.04 ± 0.03 cm/s or 1.5% ± 0.9%).

**FIGURE 6:**
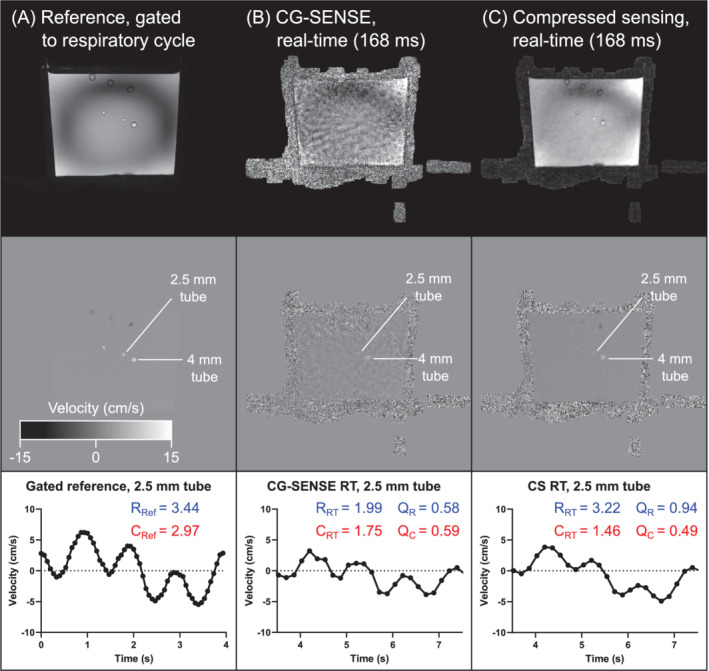
Phantom validation. Visual results showing image quality and typical flow curves. A, The reference 2D‐flow sequence, gated to the respiratory cycle of the phantom. Both the magnitude and velocity maps show good visual image quality. B, The CG‐SENSE reconstruction using 8 radial spokes per frame (temporal resolution: 168 ms). Visually, the image quality is low and the quantitative parameters *Q*_R_ and *Q*_C_ show that flow is underestimated by about 40%. C, The compressed sensing (CS) reconstruction. The respiratory variation is more accurate, with an underestimation of only 6%, whereas the cardiac oscillation is underestimated by about 50%. Data shown for 60‐bpm cardiac frequency and CS regularization factor *λ* = 10^−6^

Examples of image quality and flow curves are shown in Figure 6. The CG‐SENSE image reconstruction shows degraded image quality and underestimation of both the respiratory and cardiac amplitudes compared with the gated reference (respiratory amplitude ratio *Q*_R_ = 0.58 and cardiac amplitude ratio *Q*_C_ = 0.59). In this example, the CS real‐time reconstruction shows a smaller underestimation of the respiratory component (*Q*_R_ = 0.94), but a large underestimation of the cardiac component (*Q*_C_ = 0.49).

**FIGURE 7:**
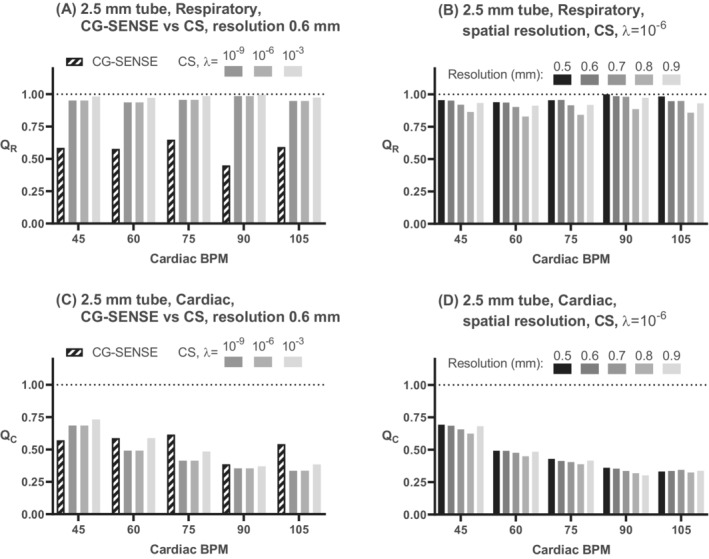
Quantitative results for respiratory and cardiac oscillations from the phantom experiment, 2.5‐mm tube. A, Comparison of CG‐SENSE and CS reconstructions with different settings of *λ* for the respiratory component ratio *Q*_R_. B, Subanalysis of *Q*_R_ for different spatial resolutions reconstructed using CS (*λ* = 10^−6^). C, Similar data as in (A), but for the cardiac component ratio *Q*_C_, and panel (D) shows the effect of spatial resolution for the cardiac ratio *Q*_C_

Quantitative results for the phantom experiments are shown in Figure 7 for the 2.5‐mm tube, and in Supporting Information Figure S2 for the 4.0‐mm tube. In this paragraph, quantitative data are given for spatial resolution 0.6 mm and *λ* = 10^−6^ unless otherwise noted, and the mean and SD are taken over all cardiac frequencies. The CG‐SENSE reconstruction underestimated both the respiratory (2.5‐mm tube: *Q*_R_ = 0.57 ± 0.07; 4.0‐mm tube: *Q*_R_ = 0.71 ± 0.18) and cardiac components (2.5‐mm tube: *Q*_C_ = 0.57 ± 0.09; 4.0‐mm tube: *Q*_C_ = 0.65 ± 0.18). The CS reconstruction showed a small underestimation of the respiratory component (2.5‐mm tube: *Q*_R_ = 0.96 ± 0.02; 4.0‐mm tube: *Q*_R_ = 0.99 ± 0.02), but underestimation of the cardiac component remained (2.5‐mm tube: *Q*_C_ = 0.46 ± 0.14; 4.0‐mm tube: *Q*_C_ = 0.64 ± 0.12). For CS reconstructions, the difference in *Q*_C_ between the 2.5‐mm and 4.0‐mm tube was statistically significant (difference 0.19 ± 0.02, *p* < 0.001).

For CS reconstructions, the cardiac component *Q*_C_ was negatively correlated with the cardiac bpm setting (2.5‐mm tube: *y* = −0.0056*x* + 0.87, *R* ^2^ = 0.87, *p* = 0.02; 4.0‐mm tube: *y* = −0.0047*x* + 1.04, *R* ^2^ = 0.89, *p* = 0.02), but not for CG SENSE. The respiratory ratio *Q*_R_ did not correlate with the bpm setting for neither CG‐SENSE nor CS reconstructions.

### 3.2 In vivo data

**FIGURE 8:**
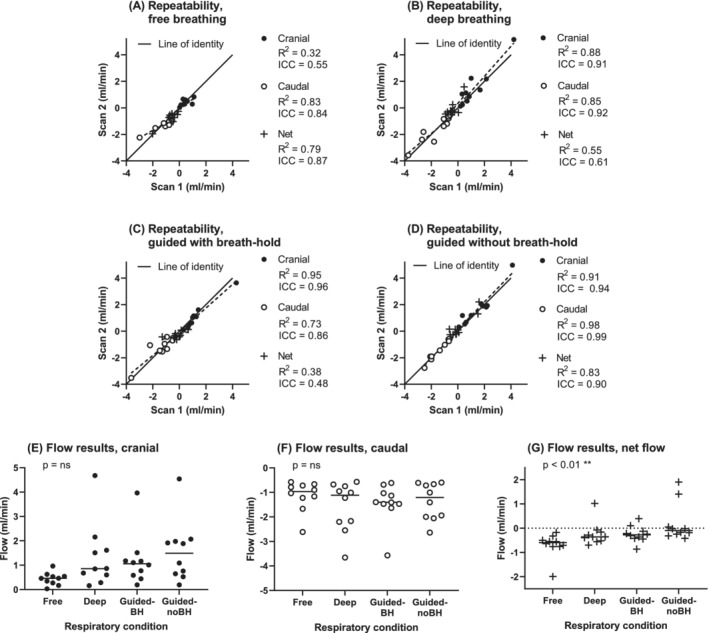
In vivo flow results. A–D, Repeatability for free breathing (A), deep breathing (B), guided with breath‐holds (C), and guided without breath‐holds (D). Regression equations and Bland–Altman bias results are given in Supporting Information Table S2. E–G, Flow data for cranial flow (E), caudal flow (F), and net flow (G). Net flow was influenced by respiratory condition (analysis of variance, *p* < 0.01), but no effect was seen for cranial or caudal flow separately (dashed line = line of regression). Abbreviation: ns, not significant

Interobserver analysis showed strong to very strong agreement for flow (ICC values ranging from 0.87 to 0.98) and a Dice coefficient of 0.80 ± 0.12 across all images. Full interobserver results are provided in Supporting Information Figure S3 and Supporting Information Table S1. Repeatability results are given in Figure 8 and Supporting Information Table S2, with moderate to very strong repeatability for cranial and caudal flow (ICC range: 0.55–0.99), and a range of weak to strong repeatability for net flow (ICC range: 0.48–0.90). Bland–Altman results for interobserver and repeatability analysis are given in Supporting Information Tables S1 and S2.

Flow measurements for the different respiratory conditions are shown in Figure 8E–G. Net flow was associated with respiratory condition (free: −0.69 ± 0.49 mL/min; deep: −0.24 ± 0.48; guide‐BH: −0.25 ± 0.34; guide‐noBH: 0.20 ± 0.79; *p* < 0.01), but there was no such effect on cranial (free: 0.44 ± 0.26 mL/min; deep: 1.34 ± 1.33; guide‐BH: 1.19 ± 1.05; guide‐noBH: 1.56 ± 1.26, *p* = ns) or caudal flow alone (free: −1.14 ± 0.61; deep: −1.58 ± 1.02; guide‐BH: −1.44 ± 0.82; guide‐noBH: −1.36 ± 0.75, *p* = not significant).

Supporting Information Figure S1D shows cross‐coherence between the respiratory bellows signal and CSF flow. There was no statistically significant difference between the respiratory conditions (free: 0.78 ± 0.24; deep: 0.67 ± 0.26; guided‐BH: 0.60 ± 0.26; guided‐noBH: 0.73 ± 0.16; *p* = 0.09).

Supporting Information Figure S4 shows flow results from one respiratory exercise in 1 subject, reconstructed with CG SENSE or CS for *λ* between 10^−9^ and 10^−2^. Quantitative flow values were stable for *λ* between 10^−3^ and 10^−9^.

### 3.3 Numerical model

**FIGURE 9:**
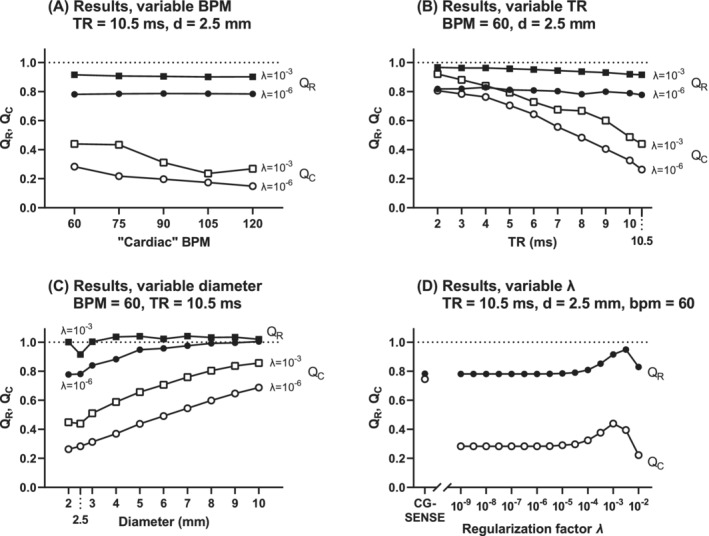
Results of numerical phantom study. A, Results for variable heart rate (bpm). B, Data for variable temporal resolution, where TR between 2 ms and 10.5 ms corresponds to a reconstructed temporal resolution between 32 ms and 168 ms. C, Dependence on aqueduct diameter. D, Dependence of the regularization parameter *λ* (horizontal axis logarithmic)

The respiratory ratio *Q*_R_ was constant for varying heart rates, whereas *Q*_C_ decreased slightly with increasing heart rate (Figure 9A). Varying temporal resolution did not affect *Q*_R_, but *Q*_C_ increased with increasing temporal resolution (Figure 9B). When increasing the aqueduct diameter, *Q*_R_ increased and approached 1 when the diameter approached 10 mm, and *Q*_C_ increased with increasing diameter (Figure 9C). The regularization factor *λ* influenced both *Q*_R_ and *Q*_C_, with higher *Q*_C_ and *Q*_R_ for *λ* = 10^−3^ compared with *λ* = 10^−6^ (Figure 9D).

## 4 DISCUSSION

This study investigated real‐time flow imaging of CSF flow in the cerebral aqueduct using golden‐angle radial MRI combined with CS image reconstruction. Phantom validation and numerical simulation of small‐diameter flow imaging showed accurate quantification of respiratory oscillations, but underestimation of cardiac oscillations. Phantom and in vivo results showed low sensitivity to the regularization factor (*λ*). In vivo repeatability was strong to very strong for cranial and caudal flow, and ranged from weak to strong for net flow. Furthermore, different respiratory conditions were associated with changes in net CSF flow in the aqueduct.

### 4.1 Accuracy and precision

The phantom experiment and numerical study show that the presented implementation of CS flow imaging can accurately capture slowly oscillating flow (eg, due to respiration) in small diameters, but cardiac oscillations are underestimated, with stronger underestimation for higher heart rates. One explanation for this may be the implicit temporal sharing of data between time frames caused by the nonlinear temporal regularization term in the image reconstruction. The effect of this is a “sliding‐window” temporal filter, which has a stronger influence on faster oscillations compared with slower ones. This is supported by the numerical model, which both shows a decrease of *Q*_C_ for increasing heart rate when TR is fixed, and an increase in *Q*_C_ for a fixed heart rate and decreasing TR (ie, improved temporal resolution). Although CS may be a useful method to accelerate other flow imaging methods such as EPI, care must be taken to validate quantitative measures.

Interestingly, underestimation of the cardiac oscillation amplitude was less evident in the larger tube (4.0‐mm diameter) compared with the smaller one (2.5‐mm diameter). This effect was confirmed in the numerical model. We speculate that because the radial acquisition heavily undersamples high spatial frequencies, flow in small‐diameter structures will be smoothed in time, as they contain a larger proportion of high spatial frequencies. Note that varying spatial resolution between 0.5 and 0.9 mm in the phantom did not significantly influence the results, suggesting that the nominal acquired spatial resolution is not the primary cause.

The CG‐SENSE reconstruction performs better in the numerical model (*Q*_R_, *Q*_C_ ≈ 0.8), compared with the phantom (*Q*_R_, *Q*_C_ ≈ 0.5), which we speculate may be explained by the absence of noise and other measurement imperfections in the numerical model. Although numerical and phantom data show similar trends overall, such as decreasing *Q*_C_ and constant *Q*_R_ for increased cardiac frequency, and increased *Q*_R_ and *Q*_C_ for increasing diameter, some quantitative differences remain, especially for the regularization parameter *λ*. Both *Q*_R_ and *Q*_C_ were relatively constant with respect to *λ* in the phantom with *Q*_R_ ≈ 1 for *λ* = 10^−3^, 10^−6^ and 10^−9^, and in vivo flow values were constant for *λ* between 10^−3^ and 10^−9^. In contrast, the numerical simulation showed *Q*_R_ ≈ 0.8 for *λ* = 10^−6^ and *Q*_R_ closer to 1 for *λ* = 10^−3^. This agrees with the fact that the optimal regularization parameters in CS algorithms may be subject‐specific and data set–specific.^47^, ^48^

Therefore, the differences in quantitative results between phantom and numerical data may be explained by the different conditions of the two experiments, such as the idealized measurement process in the numerical phantom, the difference in coil sensitivity profiles, and even the shape, texture, and edges of the imaged object, which may influence nonlinear image reconstruction methods such as CS. Taken together, we believe that direct quantitative comparisons between phantom and numerical data should be done with care, keeping these limitations in mind. An alternative explanation for the differences in *Q*_R_ between the phantom measurements and the numerical simulations may be an underestimation in the gated reference flow scan, thus biasing the comparison. However, gated 2D flow is generally considered to be accurate and precise.^49^

Both phantom and numerical data show that slow oscillations can be accurately quantified in vessels with small diameters. This may open the possibility to measure CSF flow in other narrow passages such as the subarachnoid space. Numerical results furthermore show that accuracy for faster oscillations may be improved by shortening TR (ie, improving acquired temporal resolution). However, this comes at the expense of a shortened acquisition window and an associated increase of readout bandwidth, and thus more noise. Furthermore, using TR = 2 ms is likely not feasible on a whole‐body system due to gradient limitations; thus, for unbiased quantification of cardiac oscillations in 2.5‐mm diameter at high spatial resolution, more efficient sampling schemes such as spiral imaging may be preferable.

For in vivo scans, we found strong to very strong interobserver agreement, showing that image quality is sufficient for reliable analysis of data. Furthermore, we found a range of weak to very strong repeatability of flow data. In general, net flow rates had weaker agreement than the cranial and caudal flow volumes, which is unsurprising because the net flow is computed as the difference of the almost equal cranial and caudal flow values, and is therefore more sensitive to errors such as noise and phase offsets.

Peters et al^50^ showed that respiration‐induced B_0_ fluctuations may confound flow measurements. They also show that a shortened delay between velocity encodings reduces this error, and that a delay below 20 ms gives negligible error. For EPI‐based methods, the delay is a full image acquisition (25–51 ms), whereas for our method the positive and negative velocity encodings are adjacent, and therefore the delay is only 10.5 ms. We investigated this effect in one of the healthy volunteers and found that there was no phase variation related to the respiration in stationary tissue near the aqueduct (Supporting Information Figure S5).

### 4.2 Relation to earlier studies

Early studies measured net CSF flow in the aqueduct by gating flow measurements to the cardiac cycle. Gideon et al found a net CSF flow rate of 0.69 ± 0.35 mL/min in the caudal direction.^51^ A more recent study by Forner Giner et al showed a net flow of 0.44 ± 0.28 mL/min,^52^ and Wåhlin et al found a net flow of 0.26 ± 0.20 mL/min,^53^ both in the caudal direction. A flow imaging study at 7 T showed an influence of spatial resolution on net CSF flow measurements (eg, 0.47 ± 0.23 mL/min for 0.8‐mm in‐plane resolution and 0.19 ± 0.16 mL/min for 0.2 mm).^54^ Considering the dependence on spatial resolution and potentially other imaging sequence details, the net flow observed in our data at resting conditions (0.69 ± 0.49 mL/min caudally) is consistent with data from earlier studies.

Chen et al^20^ used real‐time EPI and simultaneous multislice acquisition to quantify CSF flow with spatial resolution 2.5 mm, and Dreha‐Kulaczewski et al^26^, ^55^ used a radial flow imaging sequence similar to the one used in our study, with a spatial resolution of 1–1.2 mm, both in the spinal canal and the aqueduct. The spatial resolution in these studies may be insufficient for accurate flow measurements in the aqueduct, where the diameter is only 1–3 mm,^54^ as typically at least 4 pixels are required per vessel diameter to avoid overestimation of flow.^25^

Yildiz et al^24^ studied real‐time CSF flow in the foramen magnum using a Cartesian EPI sequence. Using a phantom setup similar to the one presented here, they found accurate measurements of both respiratory oscillations at 6 cycles/min and cardiac oscillations at 60 cycles/min. In contrast, we found an underestimation of cardiac oscillations. This can be explained by the larger vessel diameter used by Yildiz et al, which enables use of a lower spatial resolution (2.5 mm), which means that k‐space can be fully covered faster (temporal resolution = 50 ms), without the need of sharing temporal information between frames as for our method, leading to a higher effective temporal resolution. Furthermore, our phantom and numerical data showed an effect of vessel size independent of imaging resolution.

Temporal resolution also varies in previously published works. Yildiz et al^24^ used 50‐ms temporal resolution, Chen et al^20^ used 80 ms, and Dreha‐Kulaczewski et al^26^ used 135 ms. Our method reconstructs flow images at a resolution of 168 ms. However, underestimation of cardiac oscillations in phantom and numerical results shows that the actual temporal resolution of the flow data is lower, likely due to the temporal regularization. In summary, our results highlight the tradeoff between temporal and spatial resolution, and that they must be balanced for the research question at hand. If high temporal resolution is desired, study of a wider passage may be of interest, such as the foramen magnum, or the fourth ventricle, where Fultz et al^56^ studied CSF inflow and found evidence that CSF dynamics are connected to both neural and hemodynamic effects.

In our work, different modes of periodic breathing were investigated. Bhadelia et al used a pencil‐beam velocity measurement to quantify aqueduct CSF flow before, during, and after coughing, and found differences between healthy volunteers and patients with Chiari I malformation.^57^ Our method can be extended to nonperiodic respiratory conditions to further elucidate the connection between respiration and CSF flow.

Our study was performed at 7 T, in contrast to others using 3 T.^20^, ^24^, ^26^ The SNR is higher for 7 T compared with lower fields,^58^ which may enable higher temporal and spatial resolution. Disadvantages of 7 T include poor B_0_ and B_1_ homogeneity, although this is not of major importance around the aqueduct, and increased sensitivity to respiration as discussed previously. Furthermore, coil design is more challenging, and our head coil did not cover the foramen magnum. Gradient performance may also be important, such as for EPI readouts. Our radial sequence uses quite long readouts, which minimizes gradient delay issues and also has low gradient performance requirements.

### 4.3 Physiological aspects

We found that the respiratory condition influenced net flow of CSF through the aqueduct. This confirms earlier studies on respiratory effects on CSF flow,^24^, ^54^ and serves as a physiological proof‐of‐concept for the proposed imaging sequence and reconstruction for real‐time aqueduct CSF flow. However, the cranial or caudal flow alone was not correlated with the respiratory condition used.

Correlation between respiratory bellows signals and aqueduct CSF flow varied, with very strong correlation in some cases and weak in others. It has been shown that respiratory CSF flow differs between abdominal and thoracic breathing,^27^ and controlling for this may further elucidate the relation between respiration and CSF flow. Furthermore, our setup may have insufficient control of physiological parameters during the experiment. All flow measurements were performed back‐to‐back, with only short breaks in between. Therefore, we cannot exclude the possibility that an earlier respiratory exercise (eg, the first repetition “guided‐noBH”) can influence the following (the second repetition of “free breathing”) through short‐term changes in respiratory physiology or blood pressure. Furthermore, instructions to start the respiratory exercise were given only seconds before starting the scan, which means that there was no time to reach a physiological “steady state” of respiration, thoracic pressure, blood pressure, and CSF flow.

Detailed understanding of CSF flow dynamics is essential if CSF flow measurements are to be used diagnostically to identify flow disturbances or increased risk for reduced brain clearance. Therefore, it is important to understand the contributions of different physiological parameters, including breathing, heart rate, and sleep/wakefulness. Although flow of CSF in the aqueduct does not equal glymphatic clearance function, abnormal CSF movements could impair the overall glymphatic system. The hypothesis that respiratory‐induced CSF flow has a role in clearance is supported by findings linking obstructive sleep apnea to increased Amyloid β levels.^59^ Correspondingly, treatment of sleep apnea resulted in reduced Amyloid β accumulation. Our method of accurate and noninvasive measurements of respiratory influence on CSF flow could be used to elucidate the relationship between respiration and CSF flow, and potentially identify patients at higher risk for developing proteinopathic dementia.

### 4.4 Limitations

This study included only a small cohort of healthy subjects, aiming primarily to assess technical feasibility and robustness. Furthermore, respiratory conditions were only controlled through verbal and visual instructions to the volunteers, and respiratory volumes and pressures could not be assessed. To draw firm conclusions on CSF flow physiology, a protocol with greater control of physiological variables may be necessary.

## 5 CONCLUSIONS

Phantom validation and numerical simulations of flow in small diameters show that golden‐angle real‐time flow MRI with high spatial resolution reconstructed using CS can accurately quantify respiratory‐related variations of CSF flow in the cerebral aqueduct, but the implementation presented here underestimates the amplitude of cardiac oscillations. In vivo scans of aqueduct CSF flow show low interobserver variability and high scan–rescan repeatability, as well as an association between respiratory condition and net CSF flow. Flow quantification was sensitive to CS regularization parameters in numerical simulations, but not in phantoms or in vivo.

## CONFLICT OF INTEREST

Dr. Mads Andersen is an employee of Philips Healthcare. This has not influenced the design, execution, or data interpretation in the study. All other authors state that they have no conflicts of interest to disclose.

## Supporting information


**Figure S1.** Correlation of respiratory bellows signal and aqueduct CSF flow. A, The respiratory signal and CSF flow for the guided breath‐hold (BH) exercise in one of the volunteers. B, The power spectral densities of the signals, and the maximum power for the respiratory signal.* C, The cross‐coherence, with the value at the maximum from (B) extracted (in this case, 0.91, indicating a strong coherence between respiratory signal and CSF flow). D, The cross‐coherence at the dominating respiratory frequency for all experiments, grouped by respiratory condition (both repetitions).
**Figure S2.** Interobserver results. A–D, Free breathing (A), deep breathing (B), guided with breath‐holds (C), and guided without breath‐holds (D). Regression lines and R^2^ values are for all data points combined. For details, please see Supporting Information Table S1.
**Figure S3.** Quantitative results for respiratory and cardiac oscillations from the phantom experiment, 4.0 mm tube. A, Comparison of conjugate‐gradient (CG) SENSE and compressed‐sensing (CS) reconstructions with different settings of *λ* for the respiratory component ratio *Q*_R_. B, Subanalysis of *Q*_R_ for different spatial resolutions reconstructed using CS (*λ* = 10^−6^). C, Similar data as in (A), but for the cardiac component ratio *Q*_C_. D, The effect of spatial resolution for the cardiac ratio *Q*_C_.
**Figure S4.** Sensitivity to the regularization parameter *λ* for in vivo data. A, Flow values (cranial flow, caudal flow, and net flow) for reconstructions with CG‐SENSE or CS with different settings of *λ*. The flow values were stable for *λ* between 10^−3^ and 10^−9^. B, Flow curves for CG‐SENSE and CS for *λ* = 10^−3^, 10^−6^, and 10^−9^. The inset shows that flow curves for the different settings of *λ* were in close agreement. C,D, Visual image quality for CG‐SENSE and CS with *λ* = 10^−6^ respectively
**Figure S5.** Background phase. A, Real‐time flow image (magnitude). B, Zoomed‐in image of the area around the aqueduct. The aqueduct region of interest (ROI) is shown in blue, and a background ROI with stationary tissue in red. C, Respiratory curve (free breathing). D, Mean velocity in the two ROIs. Note that the aqueduct velocity shows correlation with the respiratory curve, whereas the background velocity is much lower and shows no correlation.
**Table S1.** Interobserver variability. Note: Data are shown as mean ± SD. Bias and SD are computed from scan 1, delineated by both observers.
**Table S2.** Repeatability. Note: Data are shown as mean ± SD.
