# Supplementary material for: Real‐time imaging of respiratory effects on cerebrospinal fluid flow in small diameter passageways
Source: Magn Reson Med. 2022 Apr 10;88(2):770–86. doi: 10.1002/mrm.29248 (PMC9324219; doi:10.1002/mrm.29248)
Supplement: Supplementary file 1 — Figure S1. Correlation of respiratory bellows signal and aqueduct CSF flow. A, The respiratory signal and CSF flow for the guided breath‐hold (BH) exercise in one of the volunteers. B, The power spectral densities of the signals, and the maximum power for the respiratory signal.* C, The cross‐coherence, with the value at the maximum from (B) extracted (in this case, 0.91, indicating a strong coherence between respiratory signal and CSF flow). D, The cross‐coherence at the dominating respiratory frequency for all experiments, grouped by respiratory condition (both repetitions). Figure S2. Interobserver results. A–D, Free breathing (A), deep breathing (B), guided with breath‐holds (C), and guided without breath‐holds (D). Regression lines and R2 values are for all data points combined. For details, please see Supporting Information Table S1. Figure S3. Quantitative results for respiratory and cardiac oscillations from the phantom experiment, 4.0 mm tube. A, Comparison of conjugate‐gradient (CG) SENSE and compressed‐sensing (CS) reconstructions with different settings of λ for the respiratory component ratio Q R. B, Subanalysis of Q R for different spatial resolutions reconstructed using CS (λ = 10−6). C, Similar data as in (A), but for the cardiac component ratio Q C. D, The effect of spatial resolution for the cardiac ratio Q C. Figure S4. Sensitivity to the regularization parameter λ for in vivo data. A, Flow values (cranial flow, caudal flow, and net flow) for reconstructions with CG‐SENSE or CS with different settings of λ. The flow values were stable for λ between 10−3 and 10−9. B, Flow curves for CG‐SENSE and CS for λ = 10−3, 10−6, and 10−9. The inset shows that flow curves for the different settings of λ were in close agreement. C,D, Visual image quality for CG‐SENSE and CS with λ = 10−6 respectively Figure S5. Background phase. A, Real‐time flow image (magnitude). B, Zoomed‐in image of the area around the aqueduct. The aqueduct region of interest (ROI) is show [file MRM-88-770-s001.pdf]

Real-time imaging of respiratory effects on cerebrospinal fluid flow in small diameters

Supporting Information Figures and Tables

Supporting Information Table S1: Interobserver variability. Data is shown as mean ± standard deviation (SD). Bias and standard deviation are computed from Scans 1 and 2, delineated by both observers.

		Observer 1 (ml/min)	Observer 2 (ml/min)	Average, observers 1&2 (ml/min)	Difference (ml/min)	Regression line	R^2	ICC
Free	Cranial	0.44±0.29	0.42±0.26	0.43±0.27	-0.02±0.10	y=0.83x+0.05	0.89	0.93
	Caudal	-1.13±0.62	-1.03±0.55	-1.08±0.58	0.10±0.15	y=0.85x-0.06	0.95	0.95
	Net	-0.69±0.50	-0.61±0.44	-0.65±0.47	0.08±0.13	y=0.97x-0.01	0.93	0.95
Deep	Cranial	1.34±1.33	1.29±1.26	1.31±1.29	-0.05±0.26	y=0.93x+0.04	0.96	0.98
	Caudal	-1.58±1.01	-1.48±0.98	-1.53±0.99	0.10±0.23	y=0.95x+0.01	0.95	0.97
	Net	-0.24±0.53	-0.19±0.51	-0.21±0.51	0.04±0.13	y=0.93x+0.03	0.94	0.97
Guided-BH	Cranial	1.19±1.03	1.07±0.98	1.13±1.00	-0.12±0.15	y=0.94x-0.04	0.98	0.98
	Caudal	-1.44±0.83	-1.29±0.75	-1.37±0.78	0.15±0.28	y=0.85x-0.07	0.89	0.92
	Net	-0.25±0.38	-0.22±0.32	-0.23±0.34	0.03±0.18	y=0.73x-0.04	0.77	0.87
Guided-noBH	Cranial	1.56±1.24	1.45±1.12	1.50±1.18	-0.11±0.22	y=0.89x+0.06	0.98	0.98
	Caudal	-1.36±0.73	-1.30±0.75	-1.33±0.73	0.07±0.22	y=0.98x+0.03	0.92	0.96
	Net	0.20±0.79	0.15±0.71	0.18±0.75	-0.04±0.14	y=0.98x-0.02	0.98	0.98

Supporting Information Table S2: Repeatability. Data is shown as mean ± standard deviation (SD).

		Scan 1 (ml/min)	Scan 2 (ml/min)	Average, scans 1&2 (ml/min)	Difference (ml/min)	Regression line	R^2	ICC
Free	Cranial	0.47±0.34	0.42±0.24	0.44±0.26	-0.05±0.29	y=0.40x+0.23	0.32	0.55
	Caudal	-1.11±0.76	-1.16±0.49	-1.13±0.61	-0.05±0.37	y=0.59x-0.51	0.83	0.84
	Net	-0.64±0.54	-0.75±0.47	-0.69±0.49	-0.10±0.25	y=0.78x-0.24	0.79	0.87
Deep	Cranial	1.20±1.22	1.48±1.48	1.34±1.33	0.28±0.54	y=1.13x+0.12	0.88	0.91
	Caudal	-1.58±1.09	-1.57±0.99	-1.58±1.02	0.01±0.42	y=0.84x-0.24	0.85	0.92
	Net	-0.38±0.40	-0.09±0.62	-0.24±0.48	0.28±0.42	y=1.16x+0.35	0.55	0.61
Guided-BH	Cranial	1.28±1.10	1.10±1.01	1.19±1.05	-0.18±0.25	y=0.89x-0.04	0.95	0.96
	Caudal	-1.48±0.87	-1.40±0.84	-1.44±0.82	0.08±0.46	y=0.82x-0.19	0.73	0.86
	Net	-0.20±0.50	-0.30±0.24	-0.25±0.34	-0.10±0.40	y=0.29x-0.24	0.37	0.48
Guided-noBH	Cranial	1.47±1.22	1.65±1.32	1.56±1.26	0.19±0.40	y=1.04x+0.13	0.91	0.94
	Caudal	-1.36±0.72	-1.37±0.78	-1.36±0.75	-0.02±0.13	y=1.06x+0.07	0.98	0.99
	Net	0.11±0.79	0.28±0.82	0.20±0.79	0.17±0.34	y=0.94x+0.18	0.83	0.90

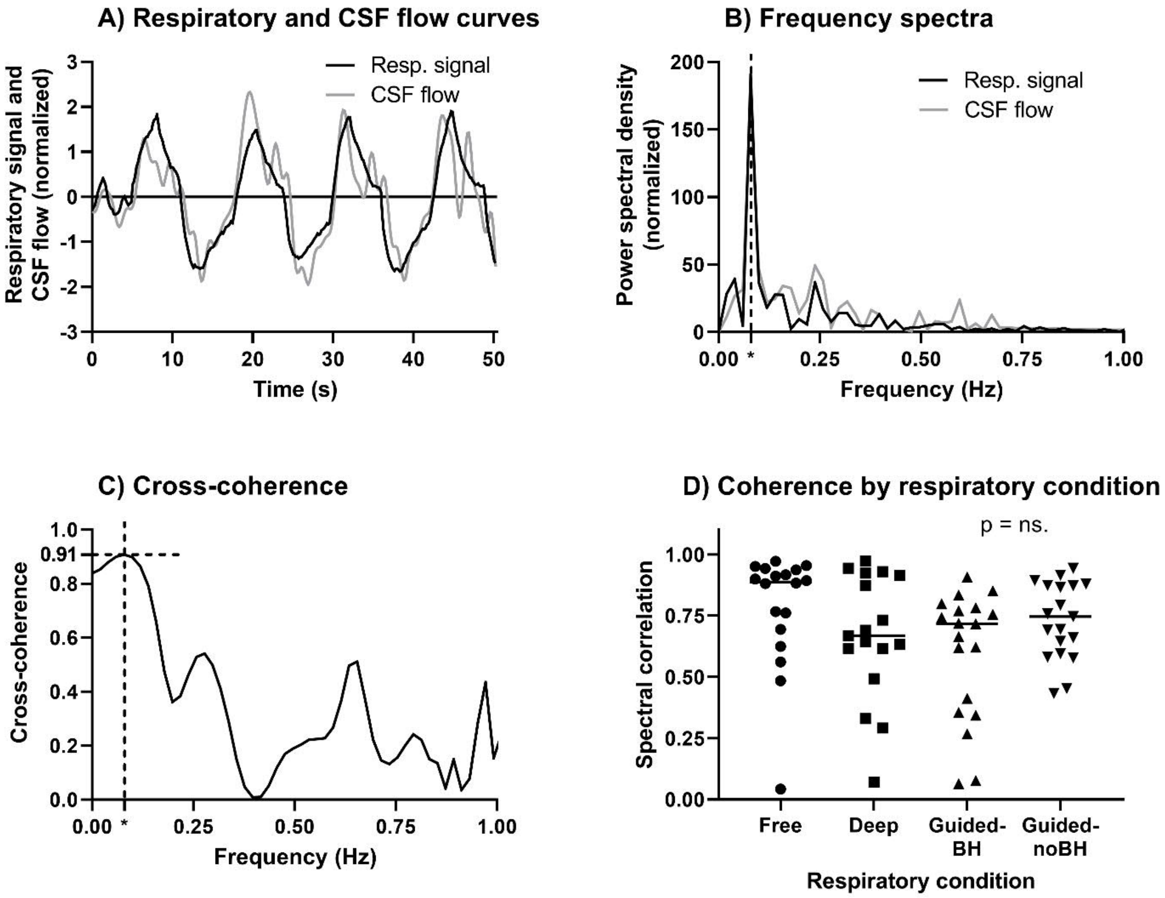

Supporting Information Figure S1: Correlation of respiratory bellows signal and aqueduct CSF flow. Panel (A) shows the respiratory signal and CSF flow for the guided-BH exercise in one of the volunteers. Panel (B) shows the power spectral densities of the signals, and the maximum power for the respiratory signal was determined (*). Panel (C) shows the cross-coherence, with the value at the maximum from Panel (B) extracted (in this case 0.91, indicating a strong coherence between respiratory signal and CSF flow). Panel (D) shows the cross-coherence at the dominating respiratory frequency for all experiments, grouped by respiratory condition (both repetitions).

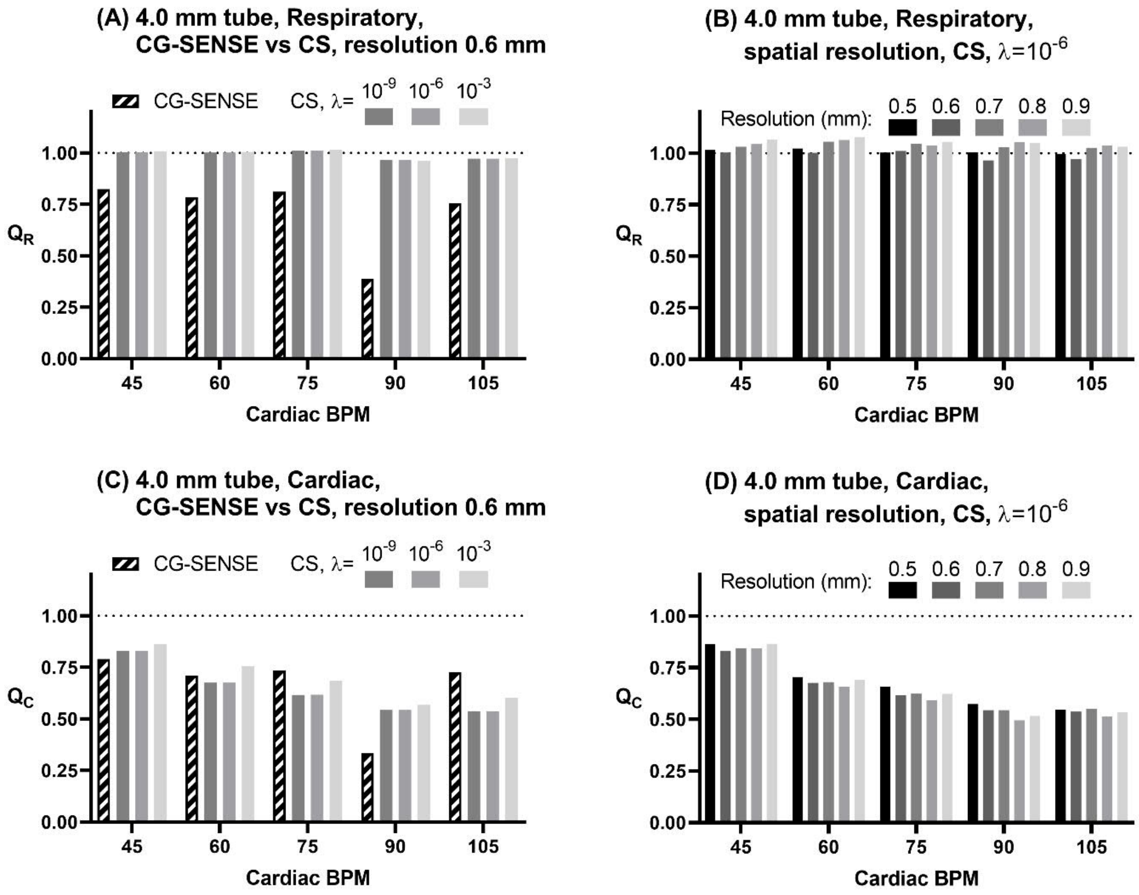

Supporting Information Figure S2: Quantitative results for respiratory and cardiac oscillations from the phantom experiment, 4.0 mm tube. Panel (A) shows a comparison of CG-SENSE and compressed sensing (CS) reconstructions with different settings of λ for the respiratory component ratio Q_R. Panel (B) shows a sub-analysis of Q_R for different spatial resolutions reconstructed using CS ($\lambda=10^{-6}$). Panel (C) shows similar data as in (A), but for the cardiac component ratio Q_C, and panel (D) shows the effect of spatial resolution for the cardiac ratio Q_C.

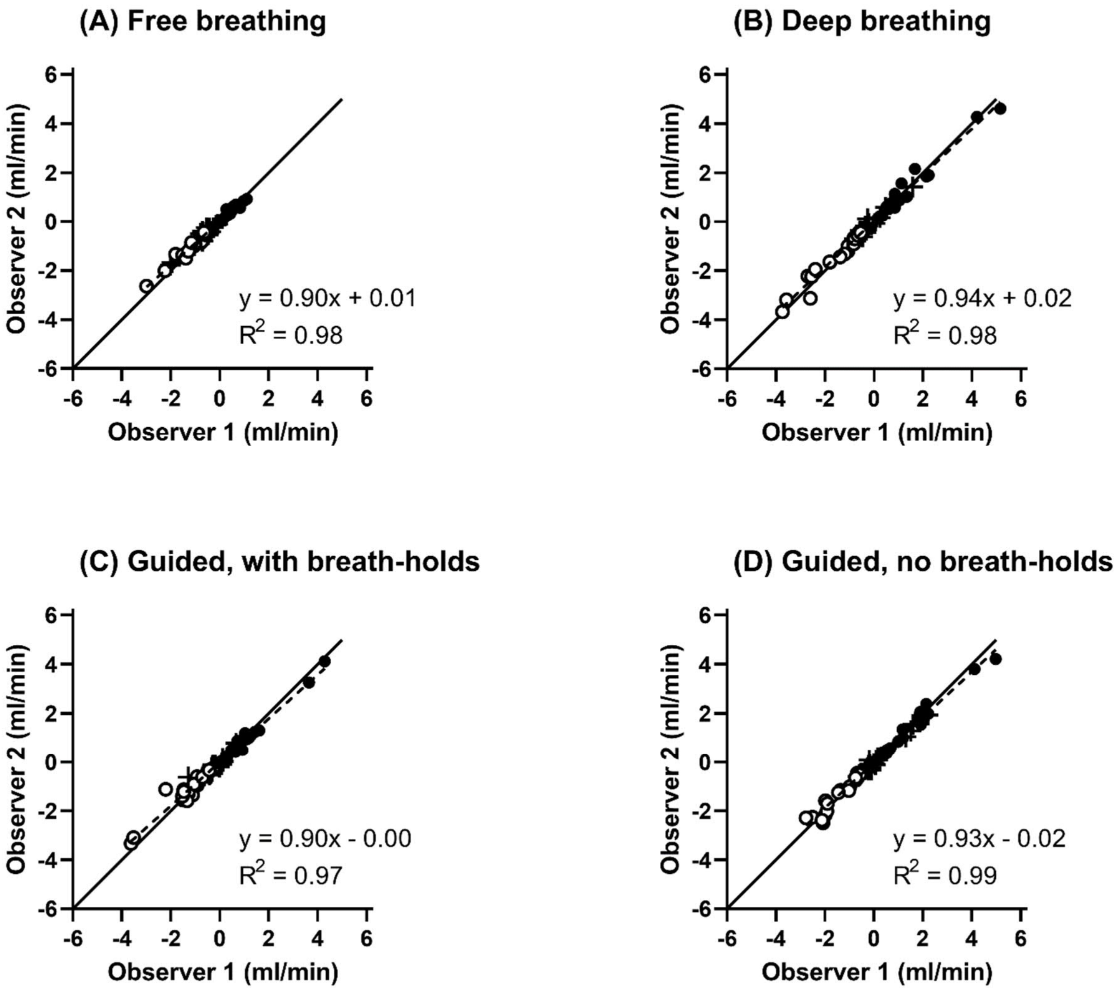

Supporting Information Figure S3: Interobserver results. Panels show: (A) free breathing, (B) deep breathing, (C) guided with breath-holds, and (D) guided without breath-holds. Regression lines and R^2 values are for all data points combined. For details, please see Supporting Information Table S1.

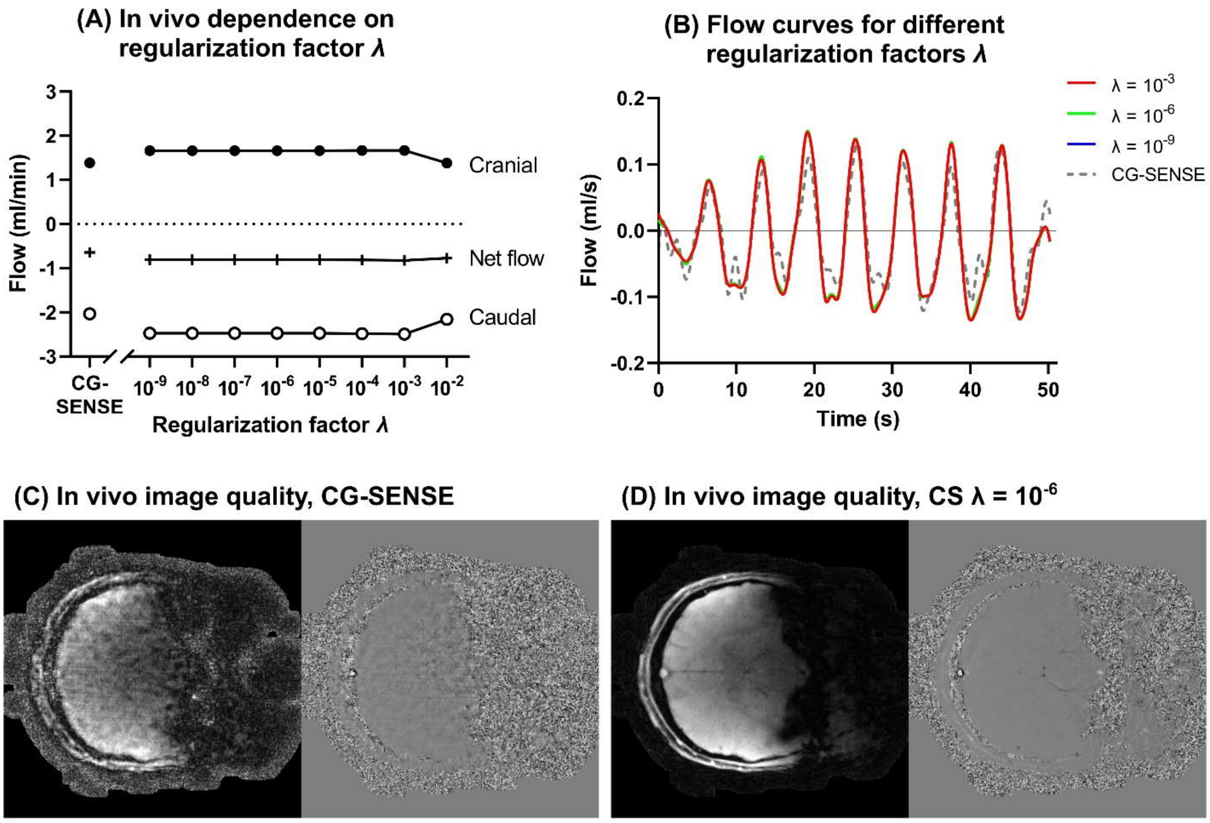

Supporting Information Figure S4: Sensitivity to the regularization parameter *λ* for in vivo data. Panel (A) shows flow values (cranial flow, caudal flow, and net flow) for reconstructions with CG-SENSE or CS with different settings of λ. The flow values were stable for λ between 10^{-3} and 10^{-9}. Panel (B) shows flow curves for CG-SENSE and CS for $\lambda = 10^{-3}$, 10^{-6} and 10^{-9} (curves are overlapping). Panels (C) and (D) show visual image quality for CG-SENSE and CS with $\lambda = 10^{-6}$ respectively.

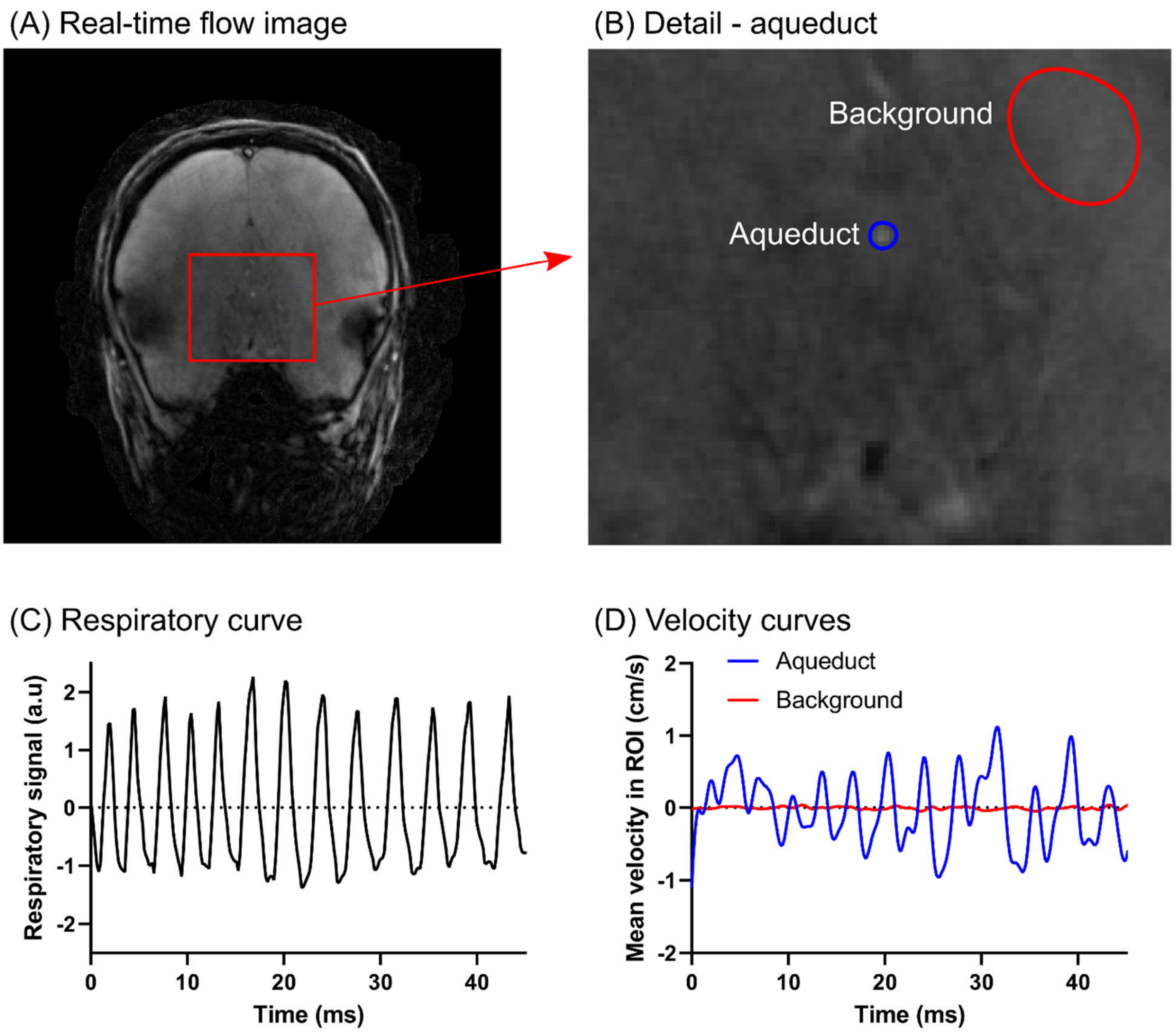

Supporting Information Figure S5: Background phase. Panel (A) shows a real-time flow image (magnitude), with Panel (B) showing a zoom to the area around the aqueduct. The aqueduct region of interest (ROI) is shown in blue, and a background ROI with stationary tissue in red. Panel (C) shows the respiratory curve (free breathing) and panel (D) the mean velocity in the two ROIs. Note that the aqueduct velocity shows correlation with the respiratory curve, while the background velocity is much lower and shows no correlation.
